# Co_3_O_4_@CoS Core-Shell Nanosheets on Carbon Cloth for High Performance Supercapacitor Electrodes

**DOI:** 10.3390/ma10060608

**Published:** 2017-06-01

**Authors:** Jinfeng Ning, Tianyu Zhang, Ying He, Congpu Jia, Petr Saha, Qilin Cheng

**Affiliations:** 1 Key Laboratory for Ultrafine Materials of Ministry of Education, School of Materials Science and Engineering, East China University of Science and Technology, Shanghai 200237, China; ningjinfeng0202@163.com (J.N.); tianyu.zhang1994@gmail.com (T.Z.); congpujia@163.com (C.J.); 2 Centre of Polymer Systems, Tomas Bata University in Zlin, nam. T. G. Masaryka 5555, Zlin 760 01, Czech Republic; saha@utb.cz

**Keywords:** Co_3_O_4_, CoS, carbon cloth, supercapacitor, nanostructured arrays

## Abstract

In this work, a two-step electrodeposition strategy is developed for the synthesis of core-shell Co_3_O_4_@CoS nanosheet arrays on carbon cloth (CC) for supercapacitor applications. Porous Co_3_O_4_ nanosheet arrays are first directly grown on CC by electrodeposition, followed by the coating of a thin layer of CoS on the surface of Co_3_O_4_ nanosheets via the secondary electrodeposition. The morphology control of the ternary composites can be easily achieved by altering the number of cyclic voltammetry (CV) cycles of CoS deposition. Electrochemical performance of the composite electrodes was evaluated by cyclic voltammetry, galvanostatic charge–discharge and electrochemical impedance spectroscopy techniques. The results demonstrate that the Co_3_O_4_@CoS/CC with 4 CV cycles of CoS deposition possesses the largest specific capacitance 887.5 F·g^−1^ at a scan rate of 10 mV·s^−1^ (764.2 F·g^−1^ at a current density of 1.0 A·g^−1^), and excellent cycling stability (78.1% capacitance retention) at high current density of 5.0 A·g^−1^ after 5000 cycles. The porous nanostructures on CC not only provide large accessible surface area for fast ions diffusion, electron transport and efficient utilization of active CoS and Co_3_O_4_, but also reduce the internal resistance of electrodes, which leads to superior electrochemical performance of Co_3_O_4_@CoS/CC composite at 4 cycles of CoS deposition.

## 1. Introduction

Today, the rapidly growing global economy has caused serious energy and environmental problems which significantly influence the development and progress of human society. Consequently, the search for sustainable and clean energy as well as efficient energy conversion and storage technologies becomes imperative [1]. Among various storage systems, supercapacitors (SCs), also known as electrochemical capacitors or ultra-capacitors, have recently attracted considerable attention due to their high power density, long cycle life, good operational safety and ultrafast charge-discharge rate [2]. Although significant progress has been made in SCs so far, the energy density is still insufficient for their practical applications. The performance of SCs depends mostly on the electrochemical activity and kinetic characteristic of the electrodes. Therefore, the design and tailoring of electrode materials with satisfactory performance are of great scientific and engineering significance.

Electrode materials for SCs mainly include carbons based on electric double layer charge storage mode, metal oxides and conducting polymers based on the pseudocapacitive charge storage mechanism [3,4]. It has been shown that the pseudocapacitance is much higher than the electric double layered capacitance due to the Faradaic redox reaction on the surface of pseudocapacitive materials [5]. As a result, various metal oxides or conducting polymers used for SCs electrodes have been investigated. Of all the metal oxides, Co_3_O_4_ is regarded as one of the most promising electrode materials because of its high specific capacitance, high redox reactivity, environmental friendliness and low cost [6]. However, the Co_3_O_4_ suffers from poor conductivity, low utilization rate, and especially low specific capacity compared to theoretical value at high current density or high scan rate, which greatly limits application when it is used as a single material [7,8]. To overcome these disadvantages, one effective strategy is to design and fabricate Co_3_O_4_ based composites combined with highly conductive materials such as carbonaceous materials [9,10,11] and conducting polymers [12,13,14] to improve the cycling stability and rate performance. The other strategy is integration of Co_3_O_4_ with metal oxides with an optimal design to enhance the electrochemical performance because of the synergistic effect between them. For instance, Cheng et al. [15] reported 3D hierarchical Co_3_O_4_@MnO_2_ core-shell heterostructures grown on nickel foam exhibited an enhanced capacitance (1693.2 F·g^−1^ at 1 A·g^−1^), long-term cycling stability (10.2% capacitance loss after 5000 cycles) and high energy density (66.2 Wh·kg^−1^ at a power density of 0.25 kW·kg^−1^) with respect to that of the individual Co_3_O_4_ nanoneedles or MnO_2_ nanosheets. Also, Gong et al. [16] developed a novel 3D nanoarchitectured Co_3_O_4_@Ni(OH)_2_ core-shell electrode presented an ultrahigh areal capacitance of 15.83 F·cm^−1^ with very high mass loading (11.9 mg·cm^−2^). Despite considerable achievements, the challenge still remains to simultaneously achieve high capacity, good rate capability and long cycling life for Co_3_O_4_ based electrodes [17,18].

Recently, transition metalsulfidessuch as CoS [19,20,21], NiS [22,23] and MoS_2_ [24] have been extensively investigated as electrode materials for SCs due to higher electrical conductivity and richer redox reactions than those of their oxide and hydroxide counterparts [25]. Thus, construction of Co_3_O_4_@CoS nanoarchitectures with a rational design is vital for boosting electrochemical performance. In particular, core-shell nanostructured arrays on conducting substrates as bind-free electrodes combine the merits of both building blocks and porous structures [26]. As a result, fast electron/ion transfer, rich accessible electroactive sites, and easy diffusion of electrolyte might be simultaneously obtained to maximize the electrochemical performance of electrodes [27,28]. In this regard, however, few studies [29] are available on the fabrication and electrochemical properties of Co_3_O_4_@CoS core-shell nanoplate heterostructures.

With the development of flexible electronics, lightweight and flexible/bendable substrates are required for practical applications. Conventional metal substrates (e.g., nickel foam, stainless steel mesh) used as a current collector inevitably increase weight of the total devices, which significantly weakened the energy density per total weight of the devices. As one of the most fascinating substrates, carbon cloth (CC) consists of a network of micro sized carbon fibers have been used as a scaffold for deposition of various active materials due to its large surface area, high physical strength, low sheet resistance, and flexible feature [30,31]. However, it is still a challenge to rationally design and fabricate Co_3_O_4_@CoS core-shell structure on CC with superior electrochemical performance by using an efficient synthetic method.

Herein, we develop a two-step electrodeposition approach to construct a 3D Co_3_O_4_@CoS core-shell nanosheet arrays grown on CC as a binder-free electrode for high-performance supercapacitor. Co_3_O_4_ nanosheet arrays are first directly grown on carbon cloth by electrodeposition, then CoS nanoparticles are electrodeposited on the surface of Co_3_O_4_ to form composite arrays. The as-prepared CC supported Co_3_O_4_@CoS composites exhibit excellent performance, such as high specific capacitances of 887.5 F·g^−1^ at a scan rate of 10 mV·s^−1^ and 764.2 F·g^−1^ at a current density of 1.0 A·g^−1^, and long-term cycling stability (78.1% capacitance retention) at 5.0 A·g^−1^ after 5000 cycles. In addition, the morphologies control of Co_3_O_4_@CoS/CC and relationship between the electrode structure and electrochemical properties are also discussed in detail.

## 2. Experimental

### 2.1. Synthesis of Co_3_O_4_/CC and CoS/CC Composites

A commercially available CC (WOS1002, CeTech Co., Ltd., Taiwan) was used as the collector and supporting substrate for the final composites. Before the experiment, five pieces of carbon cloth (2 cm × 1 cm) were treated with 10 mL aqua regia for 6 h at room temperature to remove impurities. All electrodeposition experiments were performed on an electrochemical workstation (CHI 660E, CH instruments Inc., Shanghai, China) with a standard three-electrode cell, where a CC was the working electrode, a platinum plate and an Ag/AgCl were used as the counter electrode and reference electrode, respectively. Co_3_O_4_ nanosheets were successfully grown on the treated CC substrate via a simple electrodeposition reaction and followed by an annealing treatment. In a typical process, Co(OH)_2_ precursor was electrodeposited in a 0.05 M Co(NO_3_)_2_ aqueous solution with 5% DMSO (dimethylsulfoxide). The deposition potential was –1.0 V (vs. Ag/AgCl). After 400 s of electrochemical reaction (the influence of the deposition time on surface morphology and the resulting performance of Co_3_O_4_ on carbon cloth (CC) are shown in Figures S1 and S2), the precursor was carefully washed several times with deionized water and absolute ethanol with ultrasonication, and then dried in a vacuum oven at 60 °C. Finally, the sample was calcined at 400 °C for 3 h with a heating rate of 3 °C·min^−1^ to convert to Co_3_O_4_ nanosheets.

CoS/CC composite was also obtained by electrodeposition. The reaction was performed in 0.05 M CoCl_2_·6H_2_O and 1.0 M thiourea mixed aqueous solution. The potential interval was set between −1.2 V and 0.2 V at a scan rate of 5 mV·s^−1^. The sample was produced by 4 CV cycles of CoS deposition. Finally, it was washed with ethanol and distilled water several times, and then dried in the vacuum oven at 60 °C.

### 2.2. Synthesis of Co_3_O_4_@CoS/CC Composites

The as-prepared Co_3_O_4_/CC composite acted as the working electrode. Compared to the potentiostatic electrodeposition processes, the CoS layer can be much more uniform and relatively more porous after cyclic voltammetry (CV) deposition [32]. Therefore, the thin layer of CoS particles coated on Co_3_O_4_/CC were carried out by CV on the electrochemical workstation in 0.05 M CoCl_2_·6H_2_O and 1.0 M thiourea mixed aqueous solution. The potential interval was set between −1.2 V and 0.2 V at a scan rate of 5.0 mV·s^−1^. The amount of CoS loading on the surface of Co_3_O_4_ was controlled by the number of CV cycles of CoS electrodeposition. The number of CV cycles of CoS deposition was kept at 1, 2, 3, 4, and 5, respectively. Afterwards, all the samples were washed with ethanol and distilled water several times, and then dried in the vacuum oven at 60 °C.

### 2.3. Materials Characterization

The morphology and structure of materials were tested by a field-emission scanning electron microscope (FESEM, S-4800, HITACHI, Tokyo, Japan). The chemical components of the composites were examined by X-ray photoelectron spectroscopy (XPS, ESCLAB250Xi, Thermo Scientific, Waltham, MA, USA). The structure of the samples was determined by X-ray diffraction (XRD, Cu Kα irradiation; λ = 1.5418$\dot{A}$) with a Siemens D5000 X-ray diffractometer. Raman spectra (Gloucestershire, UK) were measured with a 531.4 nm laser as the excitation source on an Acton Raman spectrometer. X-ray photoelectron spectroscopy (XPS) was collected on an AXIS Ultra DLD spectrometer (Kratos Analytical Ltd., Manchester, UK) using a monochromatized Al Kα X-ray source (1486.71 eV).

### 2.4. Electrochemical Measurements

The electrochemical measurements were carried out in a three-electrode testing system (CHI 660). The Co_3_O_4_@CoS/CC nanocomposites were used as the working electrode, an Ag/AgCl electrode and a platinum electrode were regarded as reference electrodes and counter electrodes, respectively. 1 M KOH solution was used as the electrolyte during the electrochemical reaction. Cyclic voltammetry (CV) and galvanostatic charge–discharge (GCD) curves were recorded from −0.1 to 0.5 V. The electrochemical impedance spectroscopy (EIS) were tested in the frequency from 0.01 to 100 kHz. The specific capacitance calculated from the CV curves and discharge curves can be derived from the Equations (1) and (2), respectively:

(1) $$C=\frac{\int I\cdot dV}{m\cdot \nu \cdot ∆V}$$

(2) $$C=\frac{I\cdot ∆t}{m\cdot ∆V}$$

where *I* (A) is the response of current, *m* (g) is the mass of the active materials in the electrode, *ν* (V s^−1^) is the scan rate, Δ*t* refers to the discharge time and Δ*V* (V) is the potential window. The loading mass of active materials on the electrode is around 2.06 mg.

## 3. Results and Discussion

### 3.1. Morphology and Structural Characterization

**Figure 1:**
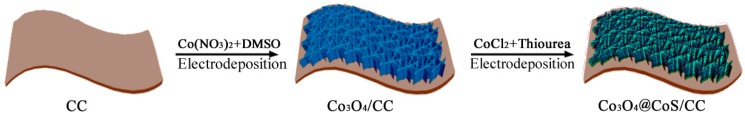
Schematic illustration of the two-step synthesis of Co_3_O_4_@CoS core-shell nanosheet arrays on carbon cloth (CC).

The preparation process of ordered Co_3_O_4_@CoS nanosheet arrays on the CC via a two-step electrodeposition method is schematically illustrated in Figure 1. Typically, Co(OH)_2_ was first electrodeposited on CC in a Co(NO_3_)_2_ aqueous solution, followed by a calcination process resulting in dense arrays of Co_3_O_4_ nanosheets grown vertically on the CC substrate; The reaction equations of Co_3_O_4_ formation process can be described as follows [33]:

(3) $${\;\mathrm{NO}}_{3}^{-}+7H_{2}O+8e^{-}\to {\mathrm{NH}}_{4}^{+}+10{\mathrm{OH}}^{-}$$

(4) $${\;\mathrm{Co}}^{2+}+2{\mathrm{OH}}^{-}\to \mathrm{Co}{\left(\mathrm{OH}\right)}_{2}$$

(5) $$\;\mathrm{Co}{\left(\mathrm{OH}\right)}_{2}\to \mathrm{CoO}+H_{2}O$$

(6) $$6\mathrm{CoO}+O_{2}\to 2{\mathrm{Co}}_{3}O_{4}$$

Subsequently, the as-synthesized Co_3_O_4_ nanosheets were coated with a thin layer of CoS particles by a controllable electrochemical deposition process to form hierarchical ternary nanocomposite. Both Co_3_O_4_ and CoS are employed as active materials, while the conductive CC as a current collector guarantees effective ion and electron transport throughout the electrode [34].

**Figure 2:**
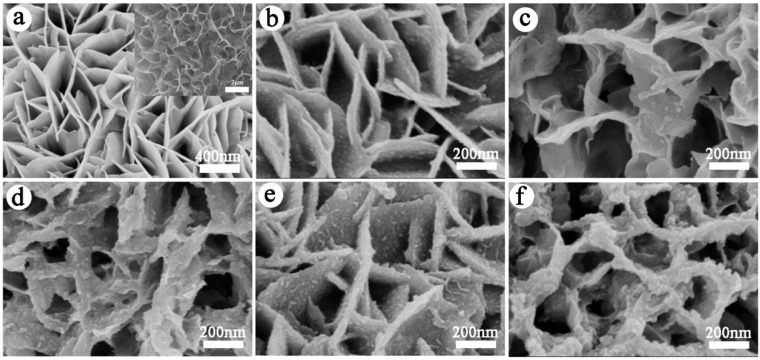
SEM images of (**a**) Co_3_O_4_/CC, inset: CoS/CC; (**b**–**f**) Co_3_O_4_@CoS/CC at 1, 2, 3, 4, 5 cycles electrodeposition, respectively.

The morphologies of porous Co_3_O_4_ nanosheet arrays and CoS-coated Co_3_O_4_ nanosheet arrays on CC were investigated by SEM and the results are presented in Figure 2. As shown in Figure 2a, the dense Co_3_O_4_ nanosheets almost grew vertically on the surface of CC. The nanosheets have an edge length of 400–500 nm and a thickness of less than 10 nm. The assembly of these sheets forms a hierarchical porous structure to facilitate penetration of electrolyte ions into the inner of electrode materials. The CoS/CC composite also exhibits the similar flake-like shape on CC (Figure 2a, inset). The surface morphology of Co_3_O_4_@CoS/CC composite with 1–5 CV cycles of CoS electrodeposition is shown in Figure 2b–f. The Co_3_O_4_ nanosheets serve as the second electrodeposition backbone for CoS particles deposition to form the core-shell nanostructures on CC. It is obvious that the smooth surface of the Co_3_O_4_ nanosheet become rough because of CoS deposition, and the thickness of nanosheets is increased to ~30 nm (Figure 2b). The composite still retains the same array structures as Co_3_O_4_/CC. With an increase in the CV cycles of CoS deposition (i.e., increase in the deposition time), it can be observed that the thickness of composite nanosheets gradually increases due to a growing amount of CoS loading on Co_3_O_4_ nanosheets (Figure 2c–e). The loading of Co_3_O_4_ on CC is about 1.06 mg, the weight gain before and after the deposition of CoS on Co_3_O_4_ /CC is the mass loading of CoS particles. With the CV cycle from 1 to 4, the mass loading of CoS increases from 0.4 to 1.0 mg. However, upon further increasing the cycles of electrodeposition, the interspaces between nanosheets are largely covered by agglomerated CoS particles (Figure 2f), which might reduce the utilization of both Co_3_O_4_ and CoS during the electrochemical reaction.

**Figure 3:**
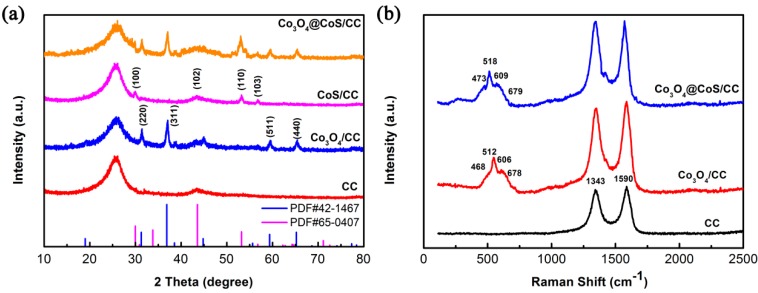
(**a**) XRD patterns; and (**b**) Raman spectra of CC and CC based composites.

The phase and structures of CC and CC based composites were confirmed by X-ray diffraction (XRD) and Raman spectra. As shown in Figure 3a, the XRD pattern of the CC displays two peaks at 2*θ* around 25° and 43° corresponding to the (002) and (100) planes of graphitic carbon, respectively. Four obvious peaks appear in the XRD pattern of Co_3_O_4_/CC are ascribed to cubic spinel Co_3_O_4_ structure phase of space group Fd3m (JCPDS No. 42-1467). After electrodeposition of CoS particles on Co_3_O_4_/CC, the new peaks at 2*θ* around 29.9°, 43.6°, 53.2° and 56.8° corresponding to (100), (102), (110), and (103) planes of CoS (JCPDS No. 65-0407) are observed in the XRD pattern of the ternary composite. The above results confirm the existence of Co_3_O_4_ and CoS in the ternary composite. In order to further study the structure of as-prepared composites, Raman spectra are carried out as shown in Figure 3b. The spectrum of the pure CC shows two characteristic peaks at 1343 and 1590 cm^−1^, corresponding to the broad D and G bands of CC [35], respectively. After growth of Co_3_O_4_@CoS core-shell nanosheets on CC, the peaks detected at around 468 cm^−1^, 512 cm^−1^, 606 cm^−1^, and 678 cm^−1^ are assigned to the E_g_, F_2g_^1^, F_2g_^2^, and A_g_^1^ modes of Co_3_O_4_ [36]. Other peaks at 473 cm^−1^, 518 cm^−1^, and 679 cm^−1^ are referred to the E_g_, F_2g_ and A_g_^1^ modes of CoS [37].

**Figure 4:**
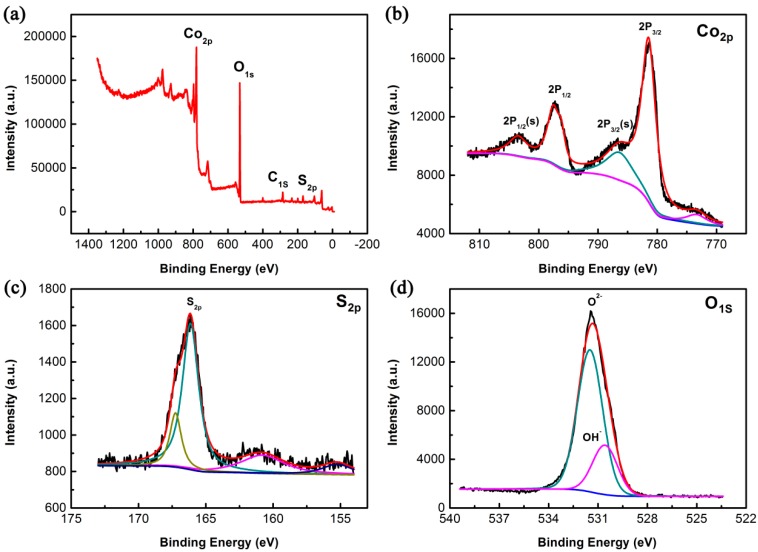
(**a**) XPS survey spectra of composites; (**b**–**d**) high-resolution XPS survey scan of Co 2p, S 2p and O 1s regions, respectively.

To verify the surface compositions of Co_3_O_4_@CoS/CC composite, XPS analysis was employed to characterize the sample. The XPS spectrum (Figure 4a) indicates the existence of C, O, Co and S elements in the ternary composite. High-resolution spectra of Co 2p, S 2p and O 1s are given in Figure 4b–d, respectively. As shown in Figure 4b, two major peaks and two satellite peaks are clearly presented in the XPS spectrum of Co 2p. The peak at 781.5 eV is related to Co 2p_3/2_, while the peak at 796.5 eV is assigned as Co 2p_1/2_. The energy difference between peaks of Co 2p_1/2_ and Co 2p_3/2_ is around 15 eV, indicating the presence of Co^2+^ and Co^3+^ [38]. Figure 4c shows the S 2p spectra with the obvious peaks at 166.5 and 161.1 eV. A binding energy of approximately 166.5 eV is a typical characteristic of S^2−^ in the composite [39]. The high-resolution XPS spectrum for O 1s in Figure 4d shows the peak at 531.3 and 530.4 eV, which is attributed to the adsorptive and lattice oxygen within Co_3_O_4_ [40]. The above XPS results identify the co-existence of Co_3_O_4_ and CoS in the ternary composites.

### 3.2. Electrochemical Properties

**Figure 5:**
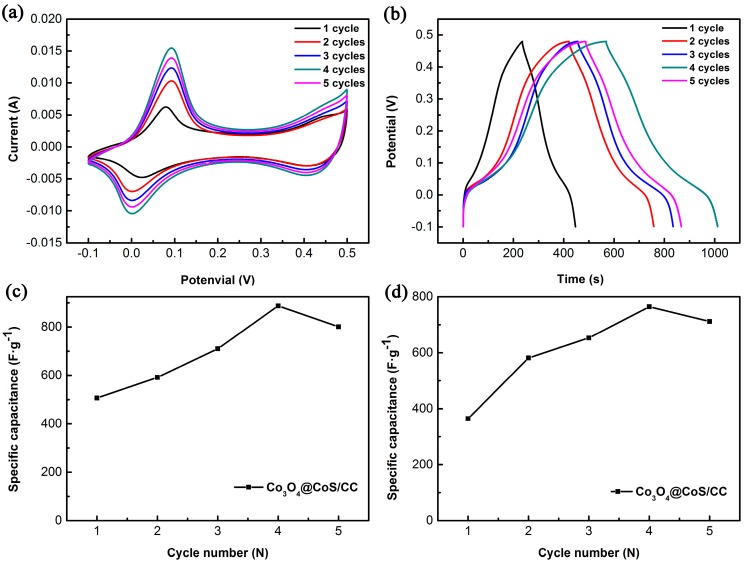
(**a**) CV curves of Co_3_O_4_@CoS/CC at different electrodeposition cycles of CoS; (**b**) Galvanostatic charge/discharge curves of Co_3_O_4_@CoS/CC at different electrodeposition cycles of CoS; (**c**) Specific capacitance value derived from (**a**) via cycle number of CoS deposition; (**d**) Specific capacitance value derived from (**b**) via cycle number of CoS deposition.

Since the Co_3_O_4_@CoS/CC core-shell nanosheet arrays with porous structures can not only offer richer redox chemistry and more electrochemical active sites, but also combine the contribution from both Co_3_O_4_ and CoS, it is expected to exhibit excellent performance as SCs electrode materials. In order to investigate the effect of CoS deposition on the capacitive performance of the ternary composite, Co_3_O_4_@CoS/CC composites synthesized with different electrodeposition cycles were measured by CV and GCD techniques. Figure 5a depicts the CV profiles of the ternary Co_3_O_4_@CoS/CC composites obtained at 1–5 cycles of CoS deposition at the scan rate of 10 mV·s^−1^. It is clear that all composite electrodes exhibit a similar CV shape with a pair of redox peaks within the potential range from −0.1 to 0.5 V. This pair of peaks is mainly due to the reversible redox reaction of Co^2+^/Co^3+^ redox couple associated with OH^−^ anions in the alkaline electrolyte, as in the case of CoS [41]. With an increase in the number of CoS electrodeposition cycles, the integral area of CV curves of composite electrodes increases to the maximum (at 4 cycles) and then decreases, indicating that Co_3_O_4_@CoS/CC composite at 4 cycles of CoS deposition possesses the largest specific capacitance. As the number of cycles increase to 5, the integral area of CV curve decreases greatly, i.e., the specific capacitance of the composite decreases dramatically. The decreasing trend of the capacitance with increasing CoS content can be explained by the surface of Co_3_O_4_ nanosheets and the open spaces between nanosheets being occupied or blocked up by the excess CoS particles, which reduces the active surface area of the composite electrode. Figure 5b presents the GCD curves of the ternary composites obtained at different cycles of CoS deposition. The longer discharge time demonstrates a larger specific capacitance; apparently, the composite obtained at 4 cycles shows the maximum capacitance. The variation tendency of capacitance is the same as the CV analysis. And the relationship between the specific capacitance (calculated from Equations (1) and (2), respectively) and cycle number of CoS deposition is plotted in Figure 5c,d. The calculating results coincide with the above conclusions from CV and GCD measurements.

**Figure 6:**
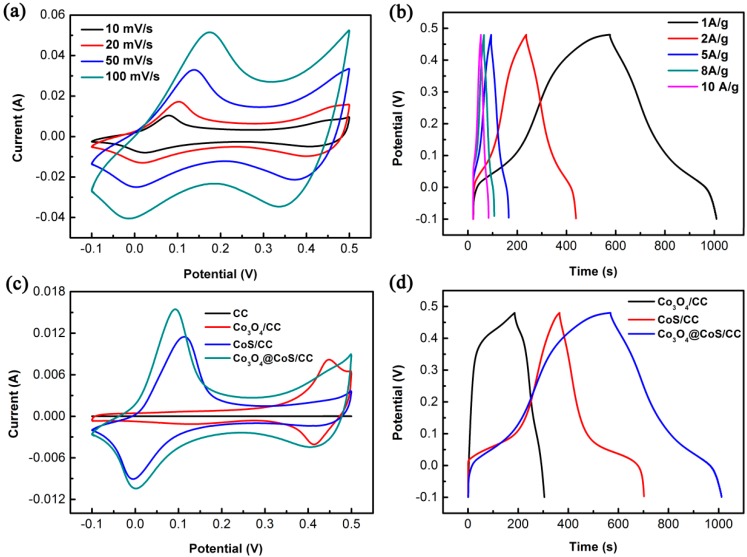
(**a**) CV curves of Co_3_O_4_@CoS/CC at different scan rates; (**b**) Galvanostatic charge/discharge curves of Co_3_O_4_@CoS/CC at different current densities; (**c**) CV curves of CC, Co_3_O_4_/CC, CoS/CC and Co_3_O_4_@CoS/CC at 10 mV·s^−1^; (**d**) Galvanostatic charge/discharge curves of Co_3_O_4_/CC, CoS/CC and Co_3_O_4_@CoS/CC at 1.0 A·g^−1^.

Since the Co_3_O_4_@CoS/CC composite at 4 cycles of CoS deposition exhibits the optimal capacitance, as a representative of composite electrodes, its electrochemical performance is evaluated in the following discussion. Figure 6a illustrates the CV curves of the Co_3_O_4_@CoS/CC composite recorded at various potential scan rates. Pairs of well-defined anodic and cathodic signals clearly appear over the entire range of scan rates from 10–100 mV·s^–1^. The current response increases with the increasing scan rate and all the curves show the similar shape, indicating rapid faradaic reaction between electrode and electrolyte, excellent electrochemical reversibility and high-rate performance of the Co_3_O_4_@CoS/CC electrode material [15]. To further evaluate the performance of the Co_3_O_4_@CoS/CC electrode, GCD measurements were performed at current densities of 1.0–10 A·g^–1^. All the curves have good symmetry but deviate from a straight and flat line, suggesting that the capacitance mainly results from faradic pseudocapacitance. Even at high current density of 10 A·g^−1^, the GCD curves have nearly symmetrical charge/discharge profiles due to the excellent interfacial contact between Co_3_O_4_@CoS core-shell nanostructures and carbon cloth, fast transportation of electrons and low equivalent series resistance.

To demonstrate the role of Co_3_O_4_ and CoS, CV curves of CC, Co_3_O_4_/CC, CoS/CC and Co_3_O_4_@CoS/CC are carried out at the same scan rate of 10 mV·s^−1^, as shown in Figure 6c. The multiple peaks at different potentials correspond to the formation of the number of cobalt oxide phases with different oxidation states [7]. The following reversible reactions (Equations (7) and (8)) take place during the redox process.

(7) $${\mathrm{Co}}_{3}O_{4}+{\mathrm{OH}}^{-}+H_{2}O\to 3\mathrm{CoOOH}+e^{-}$$

(8) $$3\mathrm{CoOOH}+e^{-}\to {\mathrm{Co}}_{3}O_{4}+{\mathrm{OH}}^{-}+H_{2}O$$

The anodic peaks (at positive current density) and cathodic peaks (at negative current density) in the CV curves are due to the oxidation and reduction processes, respectively. Two reactions of CoS are proposed for electrochemical reactions [42]:

(9) $$\mathrm{CoS}+{\mathrm{OH}}^{-}↔\mathrm{CoSOH}+H_{2}O+e^{-}$$

(10) $$\mathrm{CoSOH}+{\mathrm{OH}}^{-}↔\mathrm{CoSO}+H_{2}O+e^{-}$$

The redox peaks exist in the CV curves of the three kinds of electrode materials except for that of CC, which reflect their predominately pseudocapacitive behavior. It can be noted that the capacitance contribution of the CC substrate to the composite electrodes is very small and can be neglected owing to its very low integral area as compared to that of other composite electrodes. A comparison of the CV curves of all the composite electrodes reveals the prominent contribution of the core-shell Co_3_O_4_@CoS/CC due to the synergistic contribution of both Co_3_O_4_ and CoS pseudocapacitive materials. The enclosed area increases in the following order: CC < Co_3_O_4_/CC < CoS/CC < Co_3_O_4_@CoS/CC, indicating that the ternary composite has the largest capacitance of both binary composites, which can be confirmed by the capacitance value calculated from Equation (1). The specific capacitance of Co_3_O_4_/CC, CoS/CC and Co_3_O_4_@CoS/CC at 10 mV·s^−1^ is 295.8, 536.7, and 887.5 F·g^−1^, respectively. Figure 6d shows the GCD curves of Co_3_O_4_/CC, CoS/CC and Co_3_O_4_@CoS/CC measured at the current density of 1.0 A·g^−1^. As expected, the ternary Co_3_O_4_@CoS/CC composite electrode has the longest discharging time, that is, the largest specific capacitance which is calculated to be about 764.2 F·g^−1^ at 1.0 A·g^−1^, much higher than that of Co_3_O_4_/CC (249.2 F·g^−1^) and CoS/CC (581.1 F·g^−1^). This trend of capacitance variation is consistent with CV results, which further verifies that the ternary composite electrode yields the best capacitive performance. Such a phenomenon might be due to the following reasons. First, the deposition of CoS thin layers on the Co_3_O_4_ nanosheets results in a boost in capacitance value. Second, the combination of CC with transition metal compounds increases the conductivity, reduces charge transfer resistance and facilitates the electron transport. Third, core-shell nanosheet arrays grown on CC with open spaces not only increase the accessible surface area for the electrochemical reaction and efficient utilization of active CoS and Co_3_O_4_, but they also enable easy penetration of the electrolyte into the electrode.

**Figure 7:**
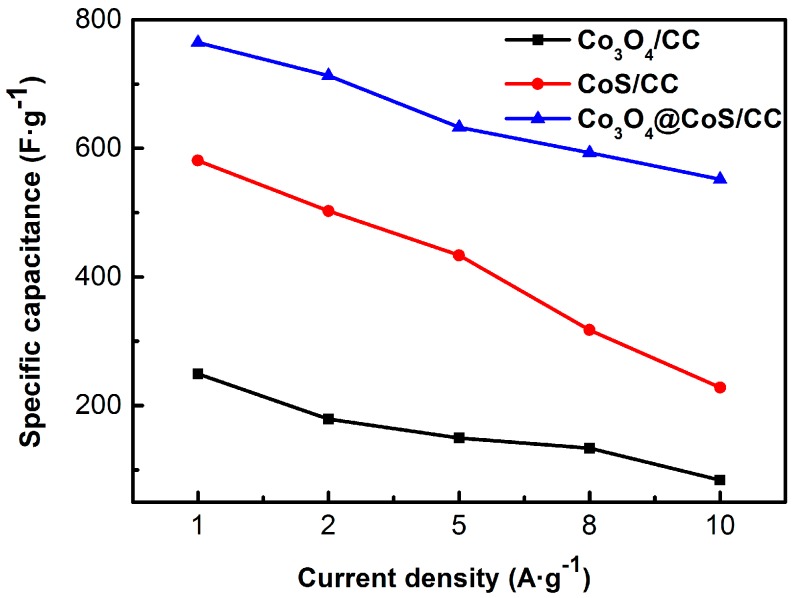
Specific capacitance curves of Co_3_O_4_/CC, CoS/CC and Co_3_O_4_@CoS/CC at different current densities.

Rate performance of composite electrodes was also studied by GCD measurements presented in Figure 7. It is evident that the specific capacitance of all composite electrodes decreases with increasing current density because of the reduced utilization of active materials caused by the increased diffusion of ions. Note that the Co_3_O_4_@CoS/CC ternary composite exhibit significantly higher capacitance than both binary ones over the entire current density range. The capacitance retention of the Co_3_O_4_@CoS/CC composite can keep as much as 72.2% as the current density increases from 1.0 to 10 A·g^−1^, while the Co_3_O_4_/CC and CoS/CC retain only 33.8% and 39.3% of the initial capacitance under the same condition, respectively. The enhanced rate capability of the Co_3_O_4_@CoS/CC composite is ascribed to the advantages of its hierarchical structure. Ultrathin CoS particles layer and Co_3_O_4_ nanosheets with small thickness can reduce the diffusion paths for electrons/ions and facilitate fast kinetics at high charge-discharge rates, ensuring the full utilization of CoS and Co_3_O_4_. Meanwhile, the CC substrates act as conductive paths not only to decrease internal resistance but also to provide robust adhesion for the Co_3_O_4_@CoS composite, and the strong synergistic effect between them also leads to improved performance.

**Figure 8:**
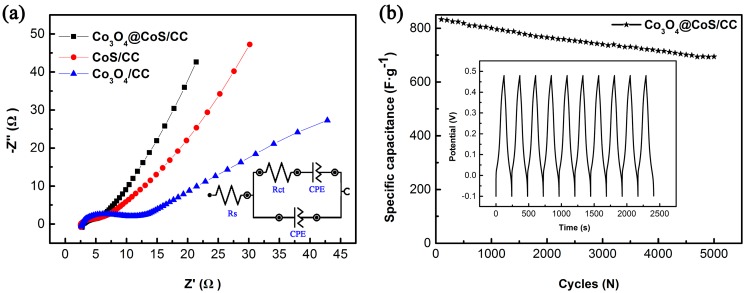
(**a**) electrochemical impedance spectroscopy (EIS) curves of CC, Co_3_O_4_/CC, and Co_3_O_4_@CoS/CC electrodes with insets showing the equivalent circuit diagram fitting the EIS; (**b**) Cycling stability of theCo_3_O_4_@CoS/CC electrode at the current density of 5 A·g^−1^ for 5000 charging-discharging cycles.

To further understand the capacitive behavior of the obtained composite electrodes, EIS were performed and the resulting Nyquist plots for Co_3_O_4_/CC, CoS/CC and Co_3_O_4_@CoS/CC electrodes are given in Figure 8a. Each electrode shows a straight line at the low-frequency region and a near semicircular shape in the high frequency region. Such a pattern of the EIS can be fitted by an equivalent circuit, as shown in the inset of Figure 8a. The intercept on the real axis represents the equivalent series resistance (*R*_s_), and the diameter of the semicircle reflects the charge transfer resistance (*R*_ct_) at the electrode/electrolyte interface [43,44]. The *R*_s_ from the Nyquist plot for Co_3_O_4_/CC, CoS/CC and Co_3_O_4_@CoS/CC electrodes is 3.29, 3.25 and 2.88 Ω, respectively, while *R*_ct_ for them is 7.40, 5.22 and 3.10 Ω respectively. The lowest *R*_s_ and *R*_ct_ of the ternary composite electrode reveal that incorporation of the CoS shell into the Co_3_O_4_ nanosheet arrays on CC improves the conductivity and decreases the charge-transfer resistance of the Co_3_O_4_@CoS/CC electrode. The redox reaction of the Co_3_O_4_@CoS/CC electrode mainly occurs on its surface and thus the capacitance primarily depends on the interfacial charge-transfer resistance. The lowest *R*_ct_ (3.1 Ω) facilitates the electron transfer and the redox reaction, thereby enhancing the capacitance. In addition, compared to both binary composite electrodes, the ternary electrode has a more vertical straight line in the low frequency range, suggesting that it has better capacitive performance due to a faster ionic diffusion rate of electrolyte ions into this ternary composite electrode. Therefore, the lower charge transfer resistance and higher ionic diffusion rate endow the Co_3_O_4_@CoS/CC composite with improved capacitance.

Long-term cycling stability of the ternary composite electrode is also carried out at the current density of 5.0 A·g^−1^ for 5000 cycles and the results are depicted in Figure 8b. The profiles of the charge and discharge curves exhibit a good electrochemical reversibility without perceptible deviations in each cycle, revealing good capacitive characteristics of the porous Co_3_O_4_@CoS/CC composite electrode. About 78.1% of its initial capacitance is retained after 5000 cycles, which is comparable to that of most Co_3_O_4_ or CoS based electrode materials (Table S1 and S2), indicating the superior cycling stability of the Co_3_O_4_@CoS/CC composite even at high current density. The above results confirm that such a rational design of Co_3_O_4_ and CoS on CC can maximize the electrochemical performance of composite electrode.

## 4. Conclusions

In summary, a facile two-step electrodeposition process has been developed for the rational design and fabrication of Co_3_O_4_@CoS core-shell nanostructures on CC for high-performance supercapacitor applications. The structural characterizations confirm that the thin CoS layers covered on the surface of Co_3_O_4_ nanosheets form 3D porous arrays on CC. A positive synergistic effect of Co_3_O_4_@CoS and CC endows the ternary composite with larger capacitance and better cycling stability than each individual binary composite. The as-prepared Co_3_O_4_@CoS/CC composite with 4 cycles of CoS deposition exhibits a high capacitance of 887.5 F·g^−1^ at 10 mV·s^−1^ and maintains 78.1% of the original capacitance after 5000 charge-discharge cycles at a large current density of 5.0 A·g^−1^. The superior electrochemical properties highlight the importance of rational design and control of electrode structures.

## Supplementary Materials

The following are available online at www.mdpi.com/1996-1944/10/6/608/s1. Figure S1: SEM images of (a) CC; (b) Co_3_O_4_-100s/CC; (c) Co_3_O_4_-200s/CC; (d) Co_3_O_4_-300s/CC; (e) Co_3_O_4_-400s/CC; (f) Co_3_O_4_-500s/CC; Figure S2: (a) CV curves of the Co_3_O_4_/CC measured at 10 mV·s^−1^; (b) Specific capacitance of the Co_3_O_4_/CC measured at 10 mV·s^−1^; (c) GCD curves of the Co_3_O_4_/CC measured at 1.0 A·g^−1^; (d) Specific capacitance of the Co_3_O_4_/CC measured at 1.0 A·g^−1^, Table S1: Comparison of the electrochemical performances of Co_3_O_4_ or CoS between literature and this study, Table S2: Comparison of the electrochemical performances and synthesis method between literature and this study.
