# Supplementary material for: Co3O4@CoS Core-Shell Nanosheets on Carbon Cloth for High Performance Supercapacitor Electrodes
Source: Materials (Basel). 2017 Jun 1;10(6):608. doi: 10.3390/ma10060608 (PMC5553425; doi:10.3390/ma10060608)
Supplement: Supplementary file 1 [file materials-10-00608-s001.pdf]

Supplementary Materials: Co_3O_4@CoS Core-Shell Nanosheets on Carbon Cloth for High Performance Supercapacitor Electrodes

Jinfeng Ning, Tianyu Zhang, Ying He, Congpu Jia, Petr Saha, Qilin Cheng

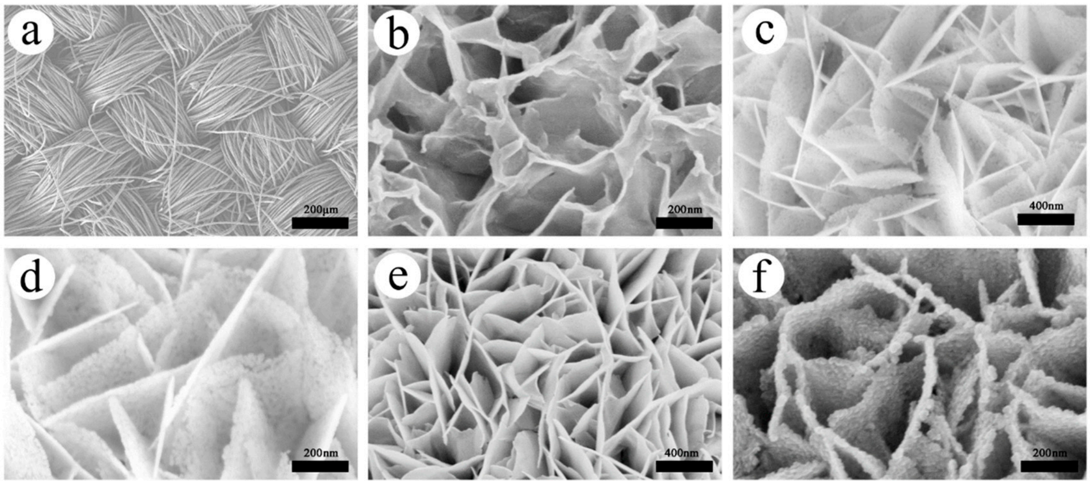

Figure S1: SEM images of (**a**) CC; (**b**) Co_3O_4-100s/CC; (**c**) Co_3O_4-200s/CC; (**d**) Co_3O_4-300s/CC; (**e**) Co_3O_4-400s/CC; (**f**) Co_3O_4-500s/CC

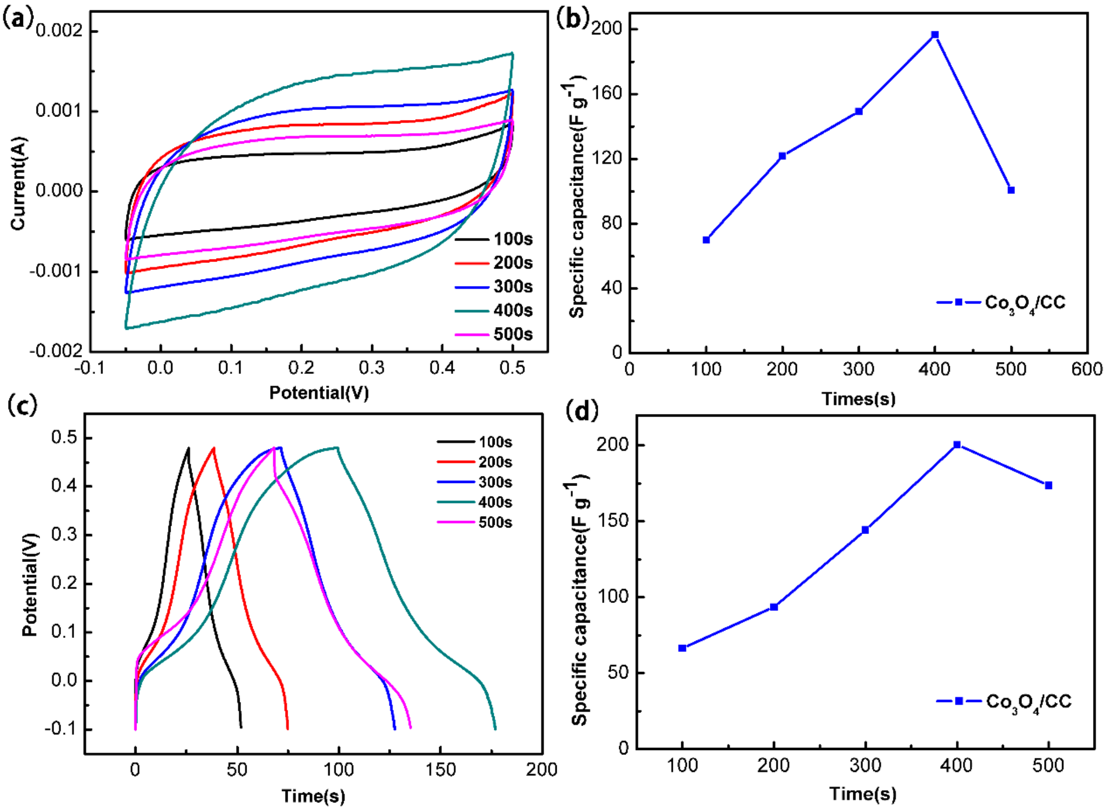

Figure S2: (**a**) CV curves of the Co_3O_4/CC measured at 10 mV·s^{-1}; (**b**) Specific capacitance of the Co_3O_4/CC measured at 10 mV·s^{-1}; (**c**) GCD curves of the Co_3O_4/CC measured at 1.0A·g^{-1}; (**d**) Specific capacitance of the Co_3O_4/CC measured at 1.0 A·g^{-1}.

Table S1: Comparison of the electrochemical performances of Co_3O_4 or CoS between literature and this study.

Materials	Maximum C_s ($F{\cdot}g^{-1}$)	Capacitance Retention	Ref.
Co_3O_4 film	162	72.2% (after 1000 cycles at 2.75 $A{\cdot}g^{-1}$)	[1]
Co_3O_4@MWCNTs	273	88% (after 1000 cycles at 0.5 $A{\cdot}g^{-1}$)	[2]
Co_3O_4@graphene	357	87% (after 1000 cycles at 1.0 $A{\cdot}g^{-1}$)	[3]
CoS nanocages	1475	88.2% (after 1000 cycles at 1.0 $A{\cdot}g^{-1}$)	[4]
CoS particulates	586	91% (after 1000 cycles at 1.0 $A{\cdot}g^{-1}$)	[5]
Co_3O_4@CoS/CC	887.5	78.1% (after 5000 cycles at 5.0 $A{\cdot}g^{-1}$)<br>96.16% (after 1000 cycles at 5.0 $A{\cdot}g^{-1}$)	This study

Table S2. Comparison of the electrochemical performances and synthesis method between literature and this study.

Materials	C_s ($F{\cdot}g^{-1}$)	Capacitance retention	Synthesis Method	Ref.
Co_3O_4@MnO_2	671	95.2% (after 2000 cycles)	Self-assembly	[6]
Co_3O_4@MnO_2/CFP	1209	80.3% (after 1000 cycles)	Hydrothermal	[7]
CoS@$NiCo_2O_4$	7.62 $F{\cdot}cm^{-1}$	71.7% (after 3000 cycles)	Hydrothermal	[8]
Co_3O_4/CF	598.9	90% (after 1000 cycles)	Electrodeposition	[9]
CoS/$Ni(OH)_2$	1837	95.8% (after 5000 cycles)	Electrodeposition	[10]
Co_3O_4@Co_3S_4/Ni Foam	425.1	93.1% (after 5000 cycles)	Hydrothermal and sulfurization	[11]
Co_3O_4@CoS/CC	887.5	78.1% (after 5000 cycles)<br>96.16% (after 1000 cycles)	Electrodeposition	This study

References

1. Tummala, R.; Guduru, R.K.; Mohanty, P.S. Nanostructured Co_3O_4 electrodes for supercapacitor applications from plasma spray technique. *J. Power* Sources **2012**, *209*, 44–51.
2. Li, T.; Li, S.; Zhang, B.; Wang, B.; Nie, D.; Chen, Z.; Yan, Y.; Wan, N.; Zhang, W. Supercapacitor electrode with a homogeneously Co_3O_4 -coated multiwalled carbon nanotube for a high capacitance. *Nanoscale Res. Lett*. 2015, *10*, 1–7.
3. Wang, H.; Shi, Y.; Li, Z.; Zhang, W.; Yao, S. Synthesisand electrochemical performance of Co_3O_4/graphene. *Chem. Res. Chin. Univ*. 2014, *30*, 650–655.
4. Jiang, Z.; Lu, W.; Li, Z.; Ho, K.H.; Li, X.; Jiao, X.; Chen, D. Synthesis of amorphous cobalt sulfide polyhedral nanocages for high performance supercapacitors. *J. Mater. Chem. A* 2014, *2*, 8603–8606.
5. Luo, F.; Li, J.; Yuan, H.; Xiao, D. Rapid synthesis of three-dimensional flower-like cobalt sulfide hierarchitectures by microwave assisted heating method for high-performance supercapacitors. *Electrochim. Acta* 2014, *123*, 183–189.
6. Che, H.; Lv, Y.; Liu, A.; Mu, J.; Zhang, X.; Bai, Y. Facile synthesis of three dimensional flower-like Co_3O_4@MnO_2 core-shell microspheres as high-performance electrode materials for supercapacitors. *Ceram. Int*. 2017, *43*, 6054–6062.
7. Wang, K.; Shi, Z.; Wang, Y.; Ye, Z.; Xia, H.; Liu, G.; Qiao, G. Co_3O_4 nanowires@MnO_2 nanolayer or nanoflakes core-shell arrays for high-performance supercapacitors: The influence of morphology on performance. *J. Alloys Compd*. 2015, *624*, 85–93.
8. Zeng, W.; Zhang, G.; Wu, X.; Zhang, K.; Zhang, H.; Hou, S.; Li, C.; Wang, T.; Duan, H. Construction of hierarchical CoS nanowire@$NiCo_2S_4$ nanosheet arrays via one-step ion exchange for high-performance supercapacitors. *J. Mater. Chem. A* 2015, *3*, 24033–24040.
9. Kazemi, S.H.; Asghari, A.; Kiani. M.A. High performance supercapacitors based on the

electrodeposited Co_3O_4 nanoflakes on electro-etched carbon fibers. *Electrochim. Acta* 2014, *138*, 9–14.

10. Li, S.; Wen, J.; Chen, T.; Xiong, L.; Wang, J.; Fang, G. In situ synthesis of 3D CoS nanoflake/$Ni(OH)_2$ nanosheet nanocomposite structure as a candidate supercapacitor electrode. *Nanotechnology* 2016, *27*, 1–9.
11. Liu, B.; Kong, D.; Zhang, J.; Wang, Y.; Chen, T.; Cheng, C.; Yang, H.Y. 3D hierarchical Co_3O_4@Co_3S_4 nanoarrays as cathode materials for asymmetric pseudocapacitors. *J. Mater. Chem. A* **2016**, *4*, 3287–3296.

© 2017 by the authors. Submitted for possible open access publication under the terms and conditions of the Creative Commons Attribution (CC BY) license (http://creativecommons.org/licenses/by/4.0/).
